# Presence of Methicillin-Resistant *Staphylococcus aureus* (MRSA) on Healthcare Workers’ Attire: A Systematic Review

**DOI:** 10.3390/tropicalmed6020042

**Published:** 2021-03-31

**Authors:** Pavlina Lena, Angela Ishak, Spyridon A Karageorgos, Constantinos Tsioutis

**Affiliations:** 1 School of Sciences, European University Cyprus, Nicosia 2404, Cyprus; p.lena@external.euc.ac.cy; 2 School of Medicine, European University Cyprus, Nicosia 2404, Cyprus; Angela.ishak.10@gmail.com (A.I.); s.karageorgos@external.euc.ac.cy (S.A.K.); 3 Department of Pediatrics, Limassol General Hospital, Limassol 3304, Cyprus

**Keywords:** MRSA, attire, clothing, contact transmission, contact precautions, laundering

## Abstract

Contaminated healthcare workers’ (HCW) clothing risk transferring methicillin-resistant *Staphylococcus aureus* (MRSA) in healthcare facilities. We performed a systematic review in Pubmed and Scopus for 2000–2020 according to the Preferred Reporting Items for Systematic Reviews and Meta-analyses (PRISMA) guidelines to analyze evidence of MRSA on HCW attire. The primary study outcome was MRSA isolation rates on HCW clothing in healthcare settings. Out of 4425 articles, 23 studies were included: 18 with 1760 HCWs, four with 9755 HCW–patient interactions and one with 512 samples. There was a notable variation in HCWs surveyed, HCW attires, sampling techniques, culture methods and laundering practices. HCW attire was frequently colonized with MRSA with the highest rates in long-sleeved white coats (up to 79%) and ties (up to 32%). Eight studies reported additional multidrug-resistant bacteria on the sampled attire. HCW attire, particularly long-sleeved white coats and ties, is frequently contaminated with MRSA. Banning certain types and giving preference to in-house laundering in combination with contact precautions can effectively decrease MRSA contamination and spread.

## 1. Introduction

Methicillin-resistant *Staphylococcus aureus* (MRSA) is a significant pathogen both in healthcare and community settings, causing a variety of infections including bloodstream infections, endocarditis, pneumonia, skin and soft tissue infections and bone and joint infections [1]. Despite a decline in its prevalence in healthcare settings worldwide primarily due to targeted efforts in the field of infection control, MRSA continues to represent a significant burden to healthcare systems and patients [2], hence its inclusion in the World Health Organization list of high priority pathogens for research and development of new antibiotics. In additional to β-lactams, MRSA strains often exhibit resistance to multiple antimicrobial classes, such as fluoroquinolones, macrolides and tetracycline [1].

Contact transmission is generally considered the most common means of transmission and direct contact occurs when microorganisms are transferred directly from one person to another [3]. Furthermore, transmission of infectious agents in healthcare settings requires three elements: a source of infectious agents, a susceptible host with a port of entry receptive to the agent and a mode of transmission for the agent. Sources of infectious agents in the healthcare setting include patients, HCWs, visitors, textiles, medical equipment and other surfaces.

A growing body of evidence suggests that HCW attire (such as scrubs and white coats) is often contaminated with microorganisms or pathogens that can cause infections or illnesses [4]. Therefore, HCW clothing may constitute a risk of transferring infections in healthcare facilities if they become contaminated. Such contamination often includes microorganisms from skin surfaces, clinical specimens such as wounds, blood samples and various excreta.

The aim of this systematic review was to collectively present and analyze all available evidence in relation to the presence of MRSA on HCW attire during routine work.

## 2. Materials and Methods

### 2.1. Search Strategy

The present systematic review was conducted in accordance with the Preferred Reporting Items for Systematic Reviews and Meta-analyses (PRISMA) guidelines [5]. PubMed and Scopus were systematically searched for the articles published from 2000 up to 28 April 2020. The search term applied consisted of the following key words: (staphylococcus OR staphylococci OR gram-positive) AND (cloth OR clothing OR textiles OR attire OR uniform OR coat OR coats), in order to identify all the published articles reporting data on the isolation of MRSA on healthcare workers’ clothing. Reference lists of final articles were also reviewed.

### 2.2. Study Selection and Quality Assessment

Two reviewers (C.T. and S.A.K.) independently determined study eligibility according to the title and the abstract of the articles. The full-text publications of the potentially relevant articles were retrieved and rescreened by the same two investigators. Disagreements were resolved by consensus with the third reviewer (A.I.). Risk of bias was assessed using the Newcastle–Ottawa scale [6] for non-randomized studies and the Rob2 tool for randomized studies [7] (Supplementary Materials, Tables S1 and S2).

### 2.3. Inclusion Criteria

Articles with the following requirements were included:

- published from 2000 onwards
- performed on humans
- evaluating the presence of MRSA on HCW attire during routine work (scrubs, uniforms, clothes, clothing apparel of physicians, nurses, students, other HCWs)
- performed in healthcare facilities (hospitals, nursing homes)
- published up to 28 April 2020
- English language of the full-text publication

### 2.4. Exclusion Criteria

Studies containing at least one of the following items were excluded:

- in vitro, animal and/or experimental
- no MRSA isolated or reported
- performed in settings other than healthcare (e.g., jails, schools, etc.)
- other surfaces, including single-use clothing (e.g., stethoscopes, gloves, single-use gowns)
- other populations (e.g., patients, visitors)
- language of the full-text publication other than English

### 2.5. Outcomes of Interest

The primary study outcome was to evaluate the rate of MRSA isolation on healthcare workers’ attire in healthcare settings. Secondary study outcomes included the methods used for sampling and isolation; the rates of MRSA isolation (prevalence or incidence of infection/colonization) in the healthcare settings under study; and other multidrug-resistant bacteria (MDRB) isolated on HCW clothing.

### 2.6. Data Extraction

Data extraction was performed by P.L. and A.I. using an extraction form in an Excel^®^ spreadsheet. The extracted data included: author, country, study period, study description, number of subjects and samples retrieved, sampling protocol used, sampling sites on HCW attire, culture method used, MRSA rates on HCW attire, other strains isolated and resistance patterns and washing protocols.

### 2.7. Definitions and Synthesis of Data

MRSA was defined as reported by study authors or if *S. aureus* strains exhibited resistance to oxacillin and/or expressed the mecA gene. HCW attire was defined as fabric clothing and apparel worn by HCWs during their routine work (e.g., white coats, uniforms, scrubs, ties, etc.).

## 3. Results

### 3.1. Literature Search

**Figure 1:**
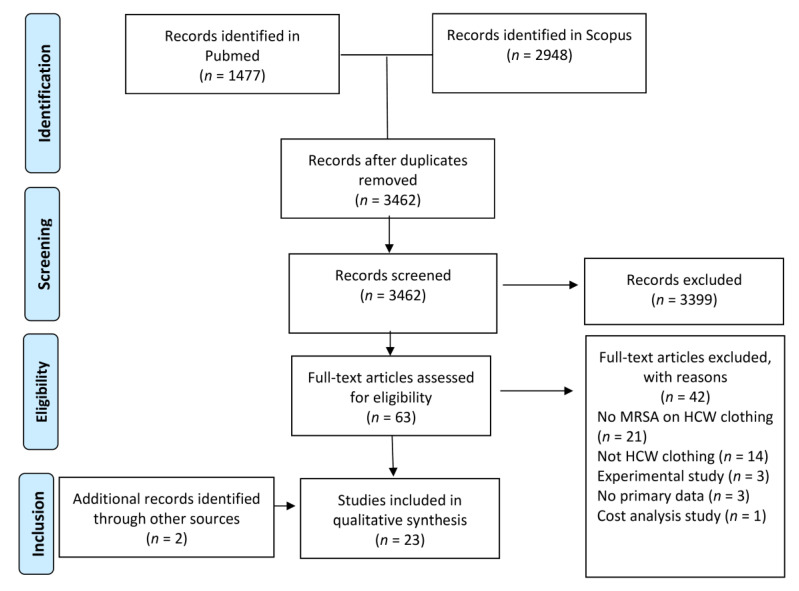
PRISMA flowchart for article screening and study selection.

A total of 4425 titles were screened from PubMed and Scopus. Following removal of duplicate studies (*n* = 963) and review of titles and abstracts, 63 articles were retrieved for full-text review. Among these, 42 studies were excluded, and 21 studies were in accordance with the inclusion criteria. Two additional articles were retrieved from references of the included studies. Finally, a total of 23 studies were incorporated in the analysis. The detailed screening process is depicted in the PRISMA flow diagram (Figure 1).

### 3.2. Study Characteristics

Table 1 summarizes characteristics of the 23 included studies. Worldwide distribution was recorded with most studies (*n* = 12) conducted in America [8,9,10,11,12,13,14,15,16,17,18,19], six—in Asia [20,21,22,23,24,25], four—in Europe [26,27,28,29] and one—in Africa [30]. Eighteen studies were cross-sectional, two were randomized controlled trials and one each of the remaining were a prospective cohort trial, a multisite prospective observational trial and a prospective cross-over trial.

### 3.3. HCW Included and Attire Sampled

A total of 1760 HCWs were included, ranging between 31 and 348 HCWs among 18 studies. In four studies, 9755 HCW–patient interactions were measured [11,13,16,18]. In one study [28], the number of participants was not mentioned, but a total of 512 samples were taken. Subjects were HCWs from various fields: nurses, care assistants and therapists, physicians, residents and medical students. Most studies sampled multiple sites from each uniform (pockets, sleeves, collar, abdominal region, waistline). Other studies sampled single specimens such as doctor’s ties [22,27,29]. Two studies examined chemically treated versus non-treated textiles [10,15]. The healthcare settings under study included hospitals, intensive care units, general wards, nursing homes and long-term care facilities (LTCF).

### 3.4. Presence of MRSA on HCW Attire

Overall, MRSA isolation rates on HCW attire ranged between 1.3% [20] and 79% [21]. However, rates varied significantly between studies, per type of attire and sampling method (Table 2). These can be summarized as follows: in the six studies evaluating gowns, MRSA rates ranged between 1.3–14%. In the five studies evaluating white coats, MRSA rates ranged between 4–79%. In the five studies evaluating scrubs, MRSA rates ranged between 0–19.1%. In the four studies evaluating uniforms (short- and long-sleeved), MRSA rates ranged between 3.5–19.1%. Finally, in the three studies evaluating MRSA isolation on ties, rates ranged between 2.5–32%.

### 3.5. Sampling Protocol and Culture Methods

Several sampling protocols were used to confirm MRSA presence on textiles (Table 2). In eight studies, the sampling time was either at the beginning of shifts [25], at the end of shifts [9,19,20,28], or both [10,14,26].

Sampling protocols varied between studies (Table 2). Fourteen studies used swabs, four used various contact plates, and one each used Rodac plates, MRSA stamp medium, gauzes instead of swabs and a Casella slit sampler. One study [15] did not specify the sampling method. Out of the 14 studies that used swabbing techniques, nine opted for enrichment of the swabs overnight and five inoculated the swabs directly on the culture media. Two studies [15,19] did not specify the culture method.

### 3.6. Reported Colonization Rates of MRSA in the Facilities under Study

Nine studies reported MRSA colonization rates in the facilities that were included in their studies (Table 2). However, these rates corresponded either to different time periods between the studies (e.g., upon admission or for the duration of the study) or to different populations (e.g., residents, nurses, patients). Jackson et al. reported patient colonization rates (nares) in the two parts of the study of 35% and 36% [18]. Koh et al., according to statistics from Malaysian hospitals, reported that 0.2–2.3% of patients were MRSA carriers [22]. Horikawa et al. reported 10% nasal carriage among 50 tested nurses [20]. Osawa et al. reported colonization prevalence in nares of HCWs of 7% and 25% (two different surveys) [21]. Treakle et al. reported 7–7.2% MRSA colonization [8]. Gaspard et al. reported prevalence rates in the three included LTCFs of 15.2%, 16% and 17.9%, respectively [28]. Finally, Roghmann et al. and Pineles et al. reported colonization of residents in the facilities of 28% and 46%, respectively [13,16].

### 3.7. Isolation of Other Multidrug-Resistant Bacteria

Nine authors reported isolation of other MDRB on HCW attire, although the exact resistance patterns were not systematically reported (Table 2). The most commonly reported MDRB were vancomycin-resistant enterococci (six studies) and multidrug-resistant *Pseudomonas aeruginosa* (2 studies).

## 4. Discussion

The aim of this systematic review was to assess all available evidence regarding isolation of MRSA on HCW attire. Our findings clearly indicate that different types of HCW attire were found to be contaminated with MRSA, which could potentially play a role in the spread of nosocomial infections. MRSA contamination rates on uniforms appeared to increase proportionally in settings with higher MRSA colonization of patients and/or HCWs. There was also variability in MRSA isolation depending on the sampling and culturing protocol used by studies; MRSA colonization rates were highest in samples where enrichment methods were used during culturing. In addition to this, the type of attire used also affected the MRSA isolation rates with studies assessing white coats having the highest MRSA contaminated uniforms. Finally, the rates seemed to be higher in the HCWs who were more likely to have patient contact, such as nurses and physicians, compared to lab personnel and students who had limited patient contact.

Consistent with a previous systematic review which suggested that white coats have a higher degree of bacterial contamination [31], our findings suggest that MRSA isolation rates were highest in white coats compared to other attire. This could be due to different laundering practices, as 70 to 100% of HCWs washed them at home every one to two weeks [8,9,17,24,30] compared to scrubs and nurse uniforms which were mainly washed using hospital services [9,10,15,28]. We could not assess whether this difference is merely due to more frequent washing or due to differences in laundering protocols as none but two [10,28] of the included studies reported their laundering technique.

There is conflicting data in the literature on whether professional laundering is more effective than home laundering in reducing bacterial contamination. The Centers for Disease Control and Prevention (CDC) recommend use of facility laundering following the Occupational Safety and Health Administration guidelines that ban using HCW attires outside healthcare settings [32]. They specify using hot-water cleaning with temperatures of over 70 °C for 25 min with a detergent that suits the attire’s fabric. On the other hand, the NHS guidance on HCW uniforms does not state a preference for domestic or professional laundering, but states specific guidelines for washing—for ten minutes at 60 °C—and recommends regular cleaning of washing machines which would be difficult to achieve with domestic machines [33]. Of note, a recent study showed that 44% of HCWs from four different hospitals in the UK did not follow the laundering protocol guidance of the NHS [34]. Our findings also show that most HCWs with the option of domestic laundering would wash their attire infrequently (less than recommended by guidelines), highlighting the need for in-house or professional laundering that would ensure recommendations are followed. This has already been done in countries such as Germany with the German Protection against Infection Act [35].

Another reason for the high contamination rates in white coats could be the length of the sleeve. Seven studies showed that white coat sleeves were highly contaminated and could also spread pathogens to other areas of the uniforms, such as pockets [8,10,12,17,23,25,30], suggesting that short-sleeved uniforms could reduce MRSA contamination rates in hospitals. Apart from the high contamination rates in uniforms, we showed that ties were also contaminated with MRSA, with all the three studies reporting that physicians rarely wash their ties [22,27,29]. The National Health Service (NHS) recommends against wearing ties due the high bacterial contamination rates found on their surface [33].

Apart from the type of uniforms, our findings also show that MRSA isolation rates in a specific setting affect proportionally the contamination rates of attire. Two studies [13,16] had higher contamination rates (14% vs. 5% and 11% vs. 1%, respectively) in the gowns worn during interactions with MRSA-positive patients compared to interactions with MRSA-negative patients. Gaspard et al. studied MRSA contamination rates in LTCFs with high MRSA colonization rates where standard precautions such as donning plastic aprons or gloves are often hard to apply [28,36,37], demonstrating lower MRSA contamination rates among HCWs who wore single-use plastic aprons and performed pocket use control (16.7% vs. <3.5%). Jackson et al. also demonstrated similar results in their predictive model where [18], in support of the relevant CDC recommendations [38], contact precautions would have yielded the highest net benefit in reduction of MRSA transmission. These observations suggest that high MRSA colonization in specific settings serves as an independent risk factor for HCW attire contamination and additional precautions are necessary.

On the other hand, three studies [20,24,25] reported MRSA isolation rates that were lower than expected according to regional or setting-specific MRSA colonization data. This could be due to various reasons. All the three studies used direct inoculation for culturing instead of enrichment, which, as mentioned below, has lower sensitivity for MRSA isolation. Among them, one study [24] tested MRSA contamination among students who are less likely to be in contact with patients, thus reducing the chances of MRSA spread to their coats and further supporting the importance of physician–patient interaction in MRSA transmission. Another study [25] had a hospital laundering service and samples were taken at the beginning of the morning shift before any patient interaction, which would undoubtedly give lower contamination of uniforms. Although detailed analysis is not possible due to high heterogeneity of methods and study participants, these findings indirectly imply that attire contamination could be related to the prevalence of MRSA in a healthcare setting. Future well-organized studies are warranted in order to evaluate the correlation between contamination rates of HCW clothing and MRSA prevalence.

This systematic review has further findings worth noting. Culturing methods varied between direct inoculation and swabbing enrichment, with studies using enrichment broths yielding higher MRSA rates. It is well-known that using an enrichment broth increases the sensitivity for MRSA detection [39], explaining the wide variation of isolation rates between studies that used enrichment and those that used direct inoculation. Furthermore, none of the studies used the EN ISO 14698-1:2003 standard [40], which provides guidance on determining biocontamination of textiles. This standard may provide harmonization in the sampling methods, making interpretation of the results more uniform and easier to compare in the future.

Certain limitations should be acknowledged. Differences in sampling and culturing methodology between studies limit the opportunity to draw meaningful conclusions with regard to MRSA contamination rates of attire. There was also variation in HCW groups included in each study and settings with different baseline MRSA colonization rates, which affect direct comparisons between studies. Finally, recommendations provided by each author could be considered of low significance, as they were based on their own observations and were affected by the limitations and bias of each individual study.

Future research should include standardizing culturing methods to enable comparisons between studies. There is also a need to study laundering techniques and their role in microbial decontamination of HCW attire. None of the included studies directly addressed potential links between HCW attire contamination and nosocomial infections. An in vitro experimental study showed that contaminated white coats can spread MDRB to pig skin [41], whereas it was further demonstrated that MRSA can be transmitted back to the skin from white coats [12]. Still, there is limited understanding of the mechanism through which adhesion and virulence affect transfer from skin to textiles and possibly back to skin.

## 5. Conclusions

Our findings indicate that HCW attire can be contaminated with MRSA regardless of the type and make, indicating a part in MRSA transmission. Current evidence shows that white coats and ties are more frequently contaminated compared to other HCW attire, suggesting against their use in healthcare settings, while wearing short-sleeved uniforms can be more beneficial. This alone seems insufficient to control MRSA spread and supports the need for additional control measures, such as contact precautions, especially in high-prevalence settings and nursing homes [42]. Additional suggestions that may help decrease the rate of MRSA contamination of HCW attire include providing physicians with specific guidelines on home laundering practices, using a hospital laundry service, wearing single-use protective aprons or gowns (as part of contact precautions), enforcing hand hygiene after every patient interaction, daily change of uniform and use of contact precautions, particularly in high-prevalence settings. Further research is needed to determine the role of contaminated HCW attire in the spread of healthcare-associated infections.

## Figures and Tables

**Table 1:** Characteristics of the 23 included studies in the systematic review.

Author (Year)	Country	Study Type	Healthcare Setting	Subjects and Samples (*n*); HCW Categories Sampled
Horikawa, 2001 [20]	Japan	Cross-sectional	Hospital	50 nurses
				150 samples
Perry, 2001 [26]	UK	Cross-sectional	Hospital wards: Renal Medicine, Renal Transplantation, Vascular Surgery, General Medicine and Obstetrics	57 nurses
				112 samples (56 pre-duty and 56 post-duty)
Osawa, 2003 [21]	Japan	Observational	Four hospital wards on two separate occasions (April 1998 and March 1999)	1. Seven physicians and seven nurses (nares, fingers, white coats, stethoscopes)
				2. Ten physicians and 14 nurses (fingers and white coats)
Ditchburn, 2006 [27]	Scotland	Cross-sectional	Hospital	40 physicians
Koh, 2009 [22]	Malaysia	Cross-sectional	Group 1 (physicians); hospitals	100 participants
			Group 2 (students); university	Physicians (50) and medical students (50)
Gaspard, 2009 [28]	France	Cross-sectional descriptive	Three geriatric long-term care facilities	512 total samples (256 samples (90 from nurses and 166 from care assistants) per zone)
Treakle, 2009 [8]	USA	Cross-sectional	Tertiary care hospital	148 participants
				38 students, 64 residents, 12 fellows and 31 attending physicians
McGovern, 2010 [9]	Ireland	Cross-sectional	Hospital	95 physicians
Uneke, 2010 [30]	Nigeria	Cross-sectional	University teaching hospital	103 physicians
Burden, 2011 [9]	USA	Prospective randomized controlled	University hospital	100 participants
				Group 1 (white coats) (*n* = 50)
				Group 2 (short-sleeved uniforms) (*n* = 50)
Wiener–Well, 2011 [23]	Israel	Cross-sectional	University hospital	135 participants
				238 samples
				75 nurses and 60 physicians
Banu, 2012 [24]	India	Cross-sectional	Tertiary medical hospital	100 participants
				83 students, 10 interns, 7 postgraduates
Bearman, 2012 [10]	USA	Prospective cross-over	ICU	Thirty-one HCWs were sampled weekly.
				Two thousand samples: 1019 study scrubs and 981 antimicrobial-impregnated scrubs (controls)
Morgan, 2012 [11]	USA	Prospective cohort	Six ICUs in a tertiary hospital	Sampling of hands and gowns reported as 585 HCW–patient interactions
				HCWs: nurses, therapists/physicians
Munoz–Price, 2012 [12]	USA	Cross-sectional	5 ICUs in a hospital	Total: 119
				White coats: 22
				Scrubs: 97
Roghmann, 2015 [3]	USA	Observational	13 community nursing homes	954 patient interactions
Williams, 2015 [14]	USA	Cross-sectional	5 ICUs	348 HCWs (252 nurses): 179 universal gowning/gloving and 169 usual care apparel
Anderson, 2017 [15]	USA	Randomized control	Medical and surgical ICUs of a tertiary care hospital	40 nurses
				2185 samples from clothing (120 shifts)
				Control group: standard cotton–polyester scrubs
				Scrub 1: scrubs with silver alloy embedded in fibers
				Scrub 2: scrubs with organosilane-based quaternary ammonium and hydrophobic fluoroacrylate copolymer emulsion
Pineles, 2017 [16]	USA	Multisite prospective observational	7 nursing homes	Interactions with MRSA-positive patients (*n* = 1543)
				Interactions with MRSA-negative patients (*n* = 1462)
Abu Radwan, 2019 [25]	Jordan	Cross-sectional	ICU—large military hospital	115 participants
				305 samples
				Nurses (58), physicians (20), resp. therapists (14), students (17), housekeepers (6)
Batista, 2019 [17]	Brazil	Cross-sectional	Hospital laboratories	100 college students
				300 samples
Jackson, 2019 [18]	USA	Cross-sectional	13 nursing home	Developmental set: 2200 interactions
			residents’ cohorts	
			1. March 2012–May 2014	Validation set: 3011 interactions
			2. Sept.2012-Jan.2016 (VA)	
Kanwar, 2019 [19]	USA	Cross-sectional	Acute care hospital	41 HCWs:
				25 (61%) nurses
				16 (39%) physicians

HCW: healthcare worker; ICU: intensive care unit; MRSA: methicillin-resistant *S. aureus*.

**Table 2:** Sampling protocols, culture methods and MRSA isolation rates in the included studies.

Author (Year)	Sampling Protocol	Culture Method	MRSA Prevalence in the Healthcare Setting under Study	Isolated MRSA Rates on HCW Clothing/HCW Categories with MRSA Isolated	Other MDRB Isolated
Horikawa, 2001 [20]	1 Swabbing nares	Direct incubation on the MSEY agar	10% of 50 tested nurses (nare swabs)	2/50 nurses with MRSA on gowns (4%)	None
	2 Swabbing of gowns after 16-h use from three areas (center of breast, belly and hip)			1.3% MRSA in 150 samples	
Perry, 2001 [26]	Uniforms sampled at start and end of shifts	Direct incubation of plates with the Columbia blood agar for MRSA detection	NR	Prior to the shift, 7/56 (12.5%)	VRE: 12/56 (21%) prior to the shift and 22/56 (39%) at end of the shift
	Casella slit sampler method for 30 sec on the front area, belt to hem			End of shift, 8/56 (14.3%)	
Osawa, 2003 [21]	Swabbing of the front lower half of ties	Direct incubation on BA	NR	1/40 (2.5%) = MRSA on ties	None
				Physicians	
Ditchburn, 2006 [27]	Ties were swept with a mannitol salt agar plater three times from neck of tie to the lower end	Direct incubation of plates	0.2–2.3% MRSA carriers (patients)	16/50 (32%) = MRSA on doctors’ ties	None
				0% on med students’ ties	
Koh, 2009 [22]	Sampling at the end of the morning shift	Swabbing enrichment	Unit 1: 15.2% Unit 2: 16% Unit 3: 17.9% (patients’ anterior nares, perineal, skin)	Waist zone: 43/256 (16.7%)	None
	Swabbing of the upper part of pockets and waistline			Pocket zone: 42/256 (16.4%)	
Gaspard, 2009 [28]	Self-swabbing of white coats: lapels, hip pockets, outer surfaces of cuffs with two passes	Swabbing enrichment	7% in non-ICU patients and 7.2% in ICU patients	6/119 (6%)	VRE—0%
Treakle, 2009 [8]	Contact with Columbia BA on the anterior surface of the lower part of the tie	Direct incubation	NR	8/95 (8.94%) of ties Physicians	VRE—not detected on any tie
McGovern, 2010 [29]	Swabbing of white coat cuffs and pocket mouths	Direct inoculation of swabs on blood agar	NR	MRSA assumed based on resistance to flucloxacillin (18/103 isolates, 17.5%)	*Pseudomonas aeruginosa* (9.6%) and GNB (19.1%); (R to norfloxacin, gentamicin, cotrimoxazole, amoxicillin/clavulanate, tetracycline, cefuroxime, ampicillin)
Uneke, 2010 [30]	Samples collected using the Rodac imprint method with BBL Rodac plates 8 h after the shift start from (1) white coats (breast pocket, mid bicep sleeve level and sleeve cuff) and (2) uniforms (breast pocket and sleeve cuffs)	Direct incubation	20% of the first 20 patients were colonized	White coats: total: 12/50 (24%): a) sleeve cuff: 4/50 (8%); b) pocket: 5/50 (10%); c) mid-biceps of sleeves: 3/50 (6%)	None
				Uniforms: total: 15/50 (30%): a) sleeve cuffs: 6/50 (12%); b) pockets: 9/50 (18%)	
Burden, 2011 [9]	Contact blood plates on different sites of white coats or scrubs (abdominal zone, sleeve ends (for white coats) and pockets (for scrubs)).	Direct incubation of plates	NR	8/238 samples (3.36%) gown cultures MRSA-positive	Not specified
Wiener–Well, 2011 [23]	Swabs were taken from four different areas of white coats (collar, pocket, sides and lapels)	Direct incubation on BA and the McConkey’s agar	NR	4/100 (4%)	None
Banu, 2012 [24]	Weekly swabbing from each leg cargo pocket and abdominal area; two swabs from each site at the beginning and end of shift (total of six samples per scrub)	Enrichment method	NR	Study scrubs: 37/1019 (3.6%)	VRE: not detected
				Control scrubs: 41/981 (4.5%)	
Bearman, 2012 [10]	Swabbing of hands (first) and gloves.	Swabbing enrichment	NR	6/152 (3.9%)	VRE (0.6%), *P. aeruginosa* (3.4%) (defined as susceptible to up to one antimicrobial classes)
	Gowns were sampled by swabbing each forearm twice and then swabbing the beltline				*Acinetobacter baumannii* (5.1%) (defined as susceptible to two or fewer antimicrobial classes) (all isolated from gowns)
Morgan, 2012 [11]	Collection of samples in five nonconsecutive days. White coats: the sleeve of the dominant hand and the front panel at the level of the abdomen	Direct imprint on TSA + % blood	NR	19% of all *S. aureus* were MRSA; 4/119 (3.36%) of scrubs	None
	Scrubs: abdominal areas				
Munoz–Price, 2012 [12]	Six sites of white coats (sleeves, the areas of two pockets, and knees)	Direct incubation of the MRSA stamp medium	7% and 25% colonization of HCW nares	1. White coats = 11/14 (79%)	None
				2. White coats = 9/24 (38%)	
Roghmann, 2015 [13]	Swabbing gowns after various interactions	Swabbing enrichment	28% resident colonization	MRSA contamination of gowns, interactions with colonized patients—14%, 5%—with negative patients	None
Williams, 2015 [14]	Swabbing of uniforms at the beginning and the end of shifts; scrubs: front top; white coats: front and cuffs	Enrichment of swabs	NR	7/346 (2%) HCWs: MRSA-positive clothing cultures	VRE—1/346 (0.28%)
Anderson, 2017 [15]	Specimens (probably swabs) from scrub sleeves, abdomen and pocket at the beginning and end of shifts (method not clearly stated)	NR	13% patients during admission	8/120 (6.7%)—MRSA contamination (present at the end of the shift)—four from the environment and four from patients	VRE—2/120 (acquired) (1.7%)
Pineles, 2017 [16]	Gloves and gowns were swabbed after patient interaction	Swabbing enrichment	46% of residents enrolled were MRSA-positive	Gowns: (a) MRSA-positive patients: 11% contamination rate (b) MRSA-negative patients: 1% contamination rate	None
	Resident screening on admission				
Abu Radwan, 2019 [25]	Beginning of the shift—three-site swabbing Long-sleeved: a. Side pocket of the dominant hand b. Abdominal area c. Terminal portion of the dominant hand sleeve Short-sleeved: a and b	Direct incubation on blood agar; confirmation with VITEK system	NR	a. Abd. Area:2 (1.7%)	None
				b. Pocket 1 (0.9%)	
				c. sleeve 1 (0.9%)	
				Total:3.5%	
Batista, 2019 [17]	Swabbing from white coats from: (a) collar (b) pockets (c) sleeves	Enrichment in the BHI broth and seeded in mannitol salt agar	NR	72/300 (24%) = MRSA (mecA gene-positive)	None
Jackson, 2019 [18]	End of workday: clothing, hands, shoes 1. Swabbing of hands, shoes 2. Premoistened gauzes for sleeve cuffs, pockets, shirt collar, waistline and external pockets of pants 3. Nares	NR	NR	12/41 (29%) total MRSA contamination with 7/41 (7%) on clothes.	None
				6/16 (37.5%) physicians with MRSA on clothing	
Kanwar, 2019 [19]	Swabbing from white coats and scrubs	Direct incubation + enrichment	Patient colonization 1. Development cohort, 35% 2.Validation cohort, 36%	HCW interaction—transmission of MRSA to gowns: (1) development cohort, 9% (190/2200); (2) validation cohort, 6% (186/3011)	None

BA: blood agar; BHI: brain heart infusion; GNB: gram-negative bacteria; MDRB: multidrug-resistant bacteria; MSEY: mannitol salt agar with egg yolk; NR: not reported; R: resistant; TSA: tryptic soy agar; VRE: vancomycin-resistant enterococci.

## Data Availability

Data is contained within the article or supplementary material.

## Supplementary Materials

The following are available online at https://www.mdpi.com/article/10.3390/tropicalmed6020042/s1, Table S1: Newcastle - Ottawa Quality Assessment Scale (adapted for cross sectional studies). Table S2: Risk-of-bias for each included randomized controlled trial.
