# Supplementary material for: Presence of Methicillin-Resistant Staphylococcus aureus (MRSA) on Healthcare Workers’ Attire: A Systematic Review
Source: Trop Med Infect Dis. 2021 Mar 31;6(2):42. doi: 10.3390/tropicalmed6020042 (PMC8103237; doi:10.3390/tropicalmed6020042)

Table S1. Newcastle - Ottawa Quality Assessment Scale (adapted for cross sectional studies).

	**Horikawa et al. 2001**	**Perry et al. 2001**	**Osawa et.al.2003**	**Ditchburn et al. 2006**	**Koh et al. 2009**	**Gaspard et.al.2009**	**Treakle et.al.2009**	**McGovern et al. 2010**	**Uneke et.al 2010**	**Winner-Well et.al..2011**	**Banu et al. 2012**	**Bearman et al. 2012**	**Morgan et.al. 2012**	**Munoz-Prize et.al.2012**	**Roghmann et.al. 2015**	**Williams et al. 2015**	**Abu Rawan & Ahmad. 2017**	**Pineless et.al. 2017**	**Batista et al. 2019**	**jackson et al. 2019**	**Kanwar et al. 2019**
1) Representativeness of the sample:<br>a) Truly representative of the average in the target population. * (all subjects or random sampling)<br>b) Somewhat representative of the average in the target population. * (non-random sampling)<br>c) Selected group of users.<br>d) No description of the sampling strategy.	*	*	*	*	*	*	*	*	*	*	*		*	*		*	*		*		*
**2)** Sample size:<br>a) Justified and satisfactory. *<br>b) Not justified.			*			*	*	*	*	*			*	*	*	*	*	*	*	*	
3) Non-respondents:<br>a) Comparability between respondents and non-respondents' characteristics is established, and the response rate is satisfactory. *<br>b) The response rate is unsatisfactory, or the comparability between respondents and non-respondents is unsatisfactory.<br>c) No description of the response rate or the characteristics of the responders and the non-responders.										*			*	*			*				
4)Ascertainment of the exposure (risk factor):<br>a) Validated measurement tool. **<br>b) Non-validated measurement tool, but the tool is available or described.*<br>c) No description of the measurement tool .	*	*	*	*	*	*	*	*	*	*	*	*	*	*	*	*	*	*	*	*	*
**5)Comparability:** (Maximum 2 stars)																					

1) The subjects in different outcome groups are comparable, based on the study design or analysis. Confounding factors are controlled.<br>a) The study controls for the most important factor (select one). *<br>b) The study control for any additional factor. *	*<br>*	*<br>*	*<br>*	*	*	*<br>*	*<br>*	*	*<br>*	*	*	*<br>*	*<br>*	*<br>*	*<br>*	*<br>*	*	*<br>*	*	*<br>*	*<br>*
6)**Outcome:** (Maximum 3 stars)<br><br>1) Assessment of the outcome:<br>a) Independent blind assessment. **<br>b) Record linkage. **<br>c) Self report. *<br>d) No description.<br>2) Statistical test:<br>a) The statistical test used to analyze the data is clearly described and appropriate, and the measurement of the association is presented, including confidence intervals and the probability level (p value). *<br>b) The statistical test is not appropriate, not described or incomplete.	**	**	**	**	**<br>*	**<br>*	**<br>*	**<br>*	**<br>*	**<br>*	**	**<br>*	**<br>*	**<br>*	**<br>*	**<br>*	**<br>*	**<br>*	**<br>*	**<br>*	**

Table S2. Risk-of-bias for each included randomized controlled trial.

(+) Low risk of bias; (?) unclear risk of bias; (–) high risk of bias

	Randomization process	Deviations from intended interventions	Missing outcome data	Measurement of the outcome	Selection of the reported result	Overall
Burden et al. (2011)	+	+	+	+	+	+
Anderson et al. (2017)	+	+	+	+	+	+

Risk-of-bias graph: judgement by review authors in each risk of bias category presented as percentage of all included studies

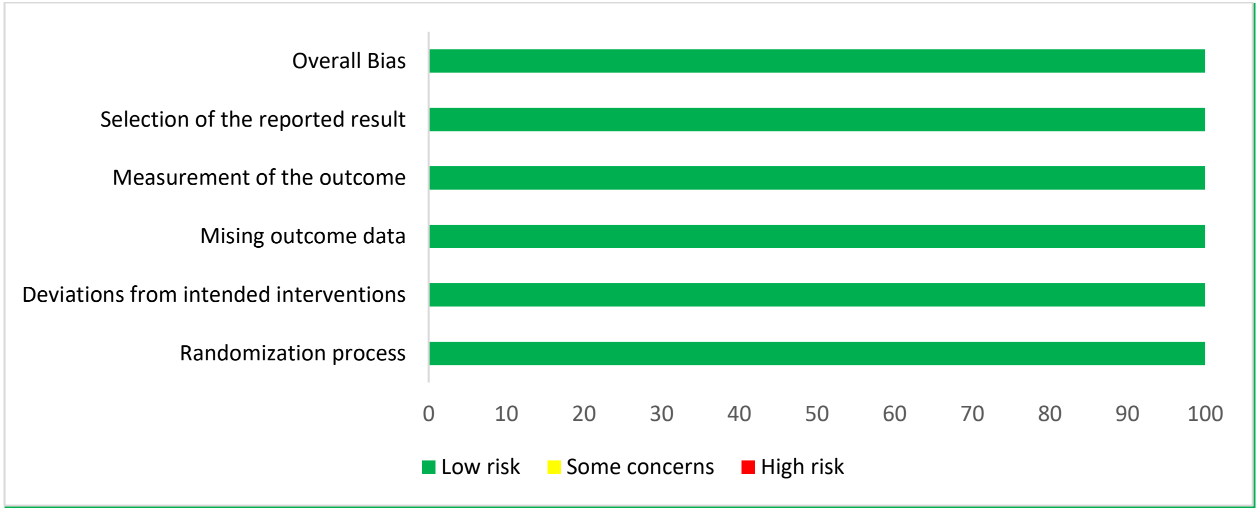

Supplement: Supplementary file 1 [file tropicalmed-06-00042-s001.pdf]
